# Getting Ready for the Dance: FANCJ Irons Out DNA Wrinkles

**DOI:** 10.3390/genes7070031

**Published:** 2016-07-01

**Authors:** Sanjay Kumar Bharti, Sanket Awate, Taraswi Banerjee, Robert M. Brosh

**Affiliations:** Laboratory of Molecular Gerontology, National Institute on Aging, National Institutes of Health, NIH Biomedical Research Center, 251 Bayview Blvd, Baltimore, MD 21224, USA; sanjay.bharti@nih.gov (S.K.B.); sanket.awate@nih.gov (S.A.); taraswi.banerjee@nih.gov (T.B.)

**Keywords:** G-quadruplex, secondary DNA structure, helicase, replication, genetic diseases, FANCJ, genomic instability, Fanconi Anemia, cancer

## Abstract

Mounting evidence indicates that alternate DNA structures, which deviate from normal double helical DNA, form in vivo and influence cellular processes such as replication and transcription. However, our understanding of how the cellular machinery deals with unusual DNA structures such as G-quadruplexes (G4), triplexes, or hairpins is only beginning to emerge. New advances in the field implicate a direct role of the Fanconi Anemia Group J (FANCJ) helicase, which is linked to a hereditary chromosomal instability disorder and important for cancer suppression, in replication past unusual DNA obstacles. This work sets the stage for significant progress in dissecting the molecular mechanisms whereby replication perturbation by abnormal DNA structures leads to genomic instability. In this review, we focus on FANCJ and its role to enable efficient DNA replication when the fork encounters vastly abundant naturally occurring DNA obstacles, which may have implications for targeting rapidly dividing cancer cells.

## 1. Dealing with G4 DNA Wrinkles

**Figure 1:**
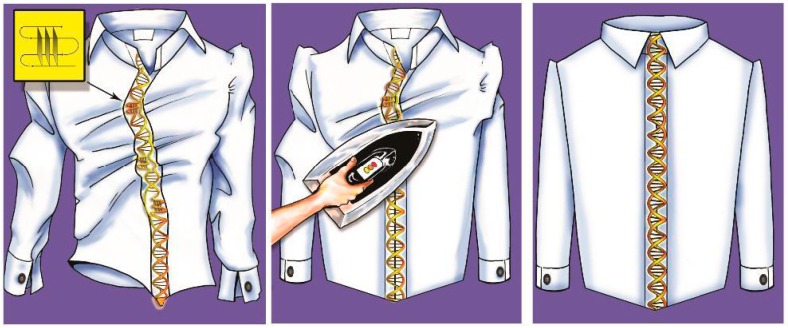
FANCJ irons out G4 DNA Wrinkles. G-quadruplex (G4) DNA structures (inset) need to be resolved by G4-interacting proteins (e.g., FANCJ helicase) to provide a suitable template for replication and other DNA metabolic processes. Once FANCJ or other G4-interacting helicases remove the G4 DNA wrinkles, the protein machinery can smoothly copy, repair, or transcribe to maintain genomic stability and cellular homeostasis.

The prom, short for promenade, is traditionally a dress-up affair in which boys wear black or white formal attire and girls wear ladies’ dresses, marking a signature social event for teenagers. The nighttime ballroom dance requires a tremendous amount of preparation that typically includes the rental of expensive tuxedos and purchase of evening gowns finely fitted and pressed for the momentous occasion. Given the investment in professional prom portraits, the prom is a night that wrinkles in one’s clothing cannot be tolerated. Such fastidious attention to wrinkle-free attire for the high school formal provides an excellent analogy for the emphasis placed on smoothing over genomic DNA to prepare for template-directed DNA synthesis that is necessary for every round of cell division (Figure 1). Resolving alternate DNA structural wrinkles that deviate from the canonical Watson–Crick DNA double helix is now considered of paramount importance to prepare for unperturbed cellular DNA replication. Secondary DNA structures that potentially influence DNA metabolism include hairpins, cruciforms, displacement loops, reversed (chicken-foot) forks, left-handed Z-DNA, triplexes, and G-quadruplexes (G4) (reviewed in [1,2]).

Of particular interest is G4 DNA, highly stable planar G-tetrad stacks that form by unconventional folding of guanine (G)-rich sequences by Hoogsteen hydrogen bonding between the bases. G4 DNA was experimentally discovered over 50 years ago by Gellert et al. using X-ray diffraction analysis of guanylic acid [3]. Over 25 years later, Sen and Gilbert determined that G-rich sequences represented by immunoglobulin switch regions or telomeric repeats form stable G4 DNA in vitro under physiological conditions [4]. Bioinformatics studies suggest that potential G4-forming sequences are highly abundant in the human genome with estimates ranging from over 300,000 to 716,000 sites [5,6,7,8]. Sequences with G4-forming potential are particularly rich at telomeres, promoter elements, ribosomal DNA, and replication origins in the nuclear genome; moreover, G4-forming sequences are also found in the mitochondrial genome where they may contribute to deletions and mutagenesis [9,10]. Although historically the existence of G4 DNA in vivo was debated, experimental results over the last decade have provided convincing evidence that G4 influences replication, transcription, and telomere DNA transactions (reviewed in [11,12]). Moreover, metabolism of G4 DNA (and G4 RNA or G4 DNA/RNA hybrids) plays prominent roles in human disease (reviewed in [13,14]). However, we are only beginning to comprehend the molecular and cellular mechanisms whereby cells deal with G-quadruplexes and other non-canonical structures that by analogy can be considered DNA wrinkles.

## 2. Experimental Evidence Implicates Human FANCJ in G4 DNA Metabolism

Specialized DNA unwinding enzymes known as helicases can resolve G4 DNA using the free energy liberated during nucleoside triphosphate binding, hydrolysis and product release (reviewed in [15,16]). Among these, the FANCJ helicase (also known as BRCA1 interacting C-terminal helicase (BACH1) or BRCA1 interacting helicase (BRIP1)) was first discovered based on its interaction with the tumor suppressor BRCA1 [17], and is implicated in hereditary breast and ovarian cancer [17,18,19] as well as the progressive bone marrow failure disease Fanconi Anemia (FA) [20,21,22]. FANCJ is a Superfamily 2 DNA helicase, which has a conserved iron-sulfur (Fe-S) cluster unique to that family that is an important structural component of the helicase core domain and is implicated in its mechanism for DNA unwinding [23,24]. FANCJ plays important roles in the FA pathway of interstrand cross-link (ICL) repair and homologous recombination (HR) repair of double-strand breaks, which has been discussed in recent reviews [25,26]. Here, we will focus on the specialized role of FANCJ to resolve alternative DNA structures that affect cellular DNA replication, transcription, and the stability of genomic DNA, including specialized DNA structures such as telomeres and other direct repeat elements.

A unique and significant role of the human Fe-S cluster DNA helicase FANCJ in G4 DNA metabolism was first evidenced by two independent experimental strategies. In human cells, FANCJ deficiency was first shown to result in a hypersensitivity to the G4 ligand Telomestatin (TMS) as evidenced by reduced proliferation, elevated apoptosis, compromised DNA synthesis, and induction of DNA damage [27]. Neither FANCA mutant or FANCD2 mutant human cell lines were sensitive to the anti-proliferative effect of TMS, suggesting that FANCJ’s function in G4 DNA metabolism in human cells is independent of its role in the classical FA pathway. Further studies showed that a deficiency in the sequence-related Fe-S cluster DNA helicases DDX11 linked to Warsaw Breakage Syndrome or XPD linked to Xeroderma pigmentosum rendered human cells sensitive to the DNA cross-linking agent mitomycin C and ultraviolet light, respectively; however, there was no apparent influence of DDX11 or XPD on DNA damage inflicted by TMS [28]. These results further suggested a specialized role of FANCJ in G4 DNA metabolism.

Consistent with the evidence that FANCJ plays a role in the replication of G4 structures, FANCJ patient cells accumulate gross chromosomal rearrangements frequently located near predicted G4-forming sequences [29]. In accord with the cell-based experiments, purified recombinant human FANCJ protein was shown to efficiently resolve in an ATP-dependent manner a variety of intermolecular G4 substrates [27,29], as well as entropically favored intramolecular G4 DNA [28], provided that a free 5′ single-stranded DNA sequence flanks the G4 structure. The strand directionality preference of FANCJ to unwind the G4 substrate is the same as that for a simple duplex DNA substrate flanked by a single-strand tail [27]; however, the molecular mechanism of FANCJ to resolve G4 DNA is unclear. Structural characterization of a sequence related Fe-S cluster XPD helicase from *Thermoplasma acidophilium* (ta), combined with a mutational and biochemical analysis, helped to elucidate the basis for 5′ to 3′ translocation directionality along single-stranded DNA [30]. The findings from this work were in agreement with those from a study that mapped taXPD protein domain contacts on DNA [31]. Together, these investigations demonstrated that the Fe-S domain of taXPD achieves a 5′ to 3′ polarity of translocation by conformational changes within the motor domain rather than an opposite orientation of single-stranded DNA binding compared to 3′ to 5′ helicases. The inability of other Fe-S helicases including human DDX11, *Escherichia coli* DinG, and taXPD to unwind unimolecular G4 DNA substrates unwound efficiently by FANCJ suggests that FANCJ is uniquely equipped to resolve G4 DNA that potentially threatens genomic stability [28].

## 3. Xenopus FANCJ Promotes Replication Past G4 DNA

Recent advancements have further improved our understanding of how the cellular machinery (e.g., FANCJ) irons out wrinkled G4 DNA. Castillo Bosch et al. demonstrated a unique requirement for a specialized DNA helicase (FANCJ) to act as a steam iron and resolve G4 DNA using Xenopus egg extract and single-stranded plasmid DNA with a G4-forming sequence at a defined position [32]. This approach enabled the authors to show convincingly that G4 structure causes DNA synthesis to pause 1–2 nucleotides away from the G4, and that depletion of FANCJ from the egg extracts resulted in a more permanent halt to DNA synthesis that could be restored by adding purified recombinant *Xenopus laevis* FANCJ protein to the cell-free system. The effect of FANCJ depletion in this assay was specific and not a general defect associated with a deficiency in the FA DNA repair pathway as suggested by the observation that depletion of another key FA protein (FANCD2) did not have an effect on DNA synthesis pausing at the G4. Aside from the technical advance of developing a cell-free system to study G-quadruplex DNA metabolism, the work helps to solidify the key role of FANCJ in resolving G-quadruplexes that impede DNA replication and potentially interfere with other G4-related processes. The Xenopus cell-free system should be amenable to manipulation and high-resolution analyses, which will hopefully provide even greater insight into the molecular mechanics of G4 resolution, the players involved, and the consequences of replication pausing for genomic stability and transcriptional regulation. However, it should be kept in mind that there may be species–specific differences in FANCJ’s role and pathways to respond to replication stress. In human cells, FANCJ cooperates with FANCD2 and BRCA2 to promote replication fork recovery independently of the FA core complex in response to the DNA polymerase inhibitor aphidicolin [33]. It is conceivable that FANCJ and its protein partners respond uniquely to pharmacological replication inhibition versus fork stalling caused by DNA secondary structure.

## 4. Studies of FANCJ and Its Role in G4 Metabolism Using Model Genetic Systems

The ability of Xenopus FANCJ to promote DNA synthesis through G4 structures suggests its functional conservation in G4 DNA metabolism, a conclusion supported by studies of FANCJ homologs in other eukaryotic systems, including the nematode DOG-1 [34,35,36] and chicken BRIP1 [37,38,39]. BRIP1-deficient DT40 cells are highly sensitive to the G4 ligand TMS [27]; furthermore, BRIP1 mutant cells exposed to TMS display elevated immunostaining by an anti-G4 monoclonal antibody, providing evidence that the FANCJ homolog is required to prevent G4 accumulation in vertebrate cells [40]. However, embryonic fibroblasts from Fancj^−/−^ mice are not sensitive to several G4 ligands, including TMS; moreover, telomeric G4 DNA sequences are stable as well, leaving doubt if murine FANCJ plays a major role in G4 DNA metabolism [41]. Instead, experimental evidence suggests that the Fe-S helicase RTEL1, which can resolve G4 DNA in vitro [42], may play a more prominent role [41,43,44].

Because some but not all DNA helicases can resolve G4 in vitro, researchers have turned to model genetic organisms to assess their unique or overlapping biological roles. For example, the WRN and BLM helicases resolve G-quadruplex substrates in vitro, but with the opposite polarity to that of FANCJ [45,46,47]. In *Caenorhabditis elegans* it was shown that loss of the BLM ortholog (HIM-6) caused reduced viability in the absence of DOG-1 accompanied by a massive increase in G-tract deletions, suggesting that FANCJ and BLM may have an overlapping function in resolving the G-rich secondary structures or that one helicase compensates for the loss of the other to some degree [36]. In contrast to what was observed for HIM-6, loss of the WRN ortholog (WRN-1) had no effect on cell viability in the absence of DOG-1 [36]. Absence of both RTEL-1 and DOG-1 resulted in synthetic lethality, which may be attributed to the requirement of RTEL-1 to inhibit inappropriate HR and thus prevent gross chromosomal rearrangements [48]. Loss of DOG-1 in the absence of RTEL-1 led to massive accumulation of Rad51 foci, an indication of uninhibited HR, which would contribute to mitotic catastrophe. As the authors suggested, the observed phenotypes may be due to the overlapping roles of RTEL-1 and DOG-1 to resolve complex G-rich DNA structures at telomeres and other specialized regions of the nematode genome. Tarailo-Graovac et al. investigated the incidence of chromosomal abnormalities observed in the absence of both DOG-1 and MDF-1, a component of the spindle-assemble checkpoint that operates to prevent abnormal whole chromosome gains and losses. Using a whole genomics approach, the authors showed that >20 base pair insertions/deletions were most commonly observed near the G-rich DNA in the absence of both DOG-1 and MDF-1, leading the authors to propose that only the neutral or advantageous insertions/deletions were tolerated in this genetic background after longtime propagation [49].

Loss of DOG-1 can cause an increase in unresolved G4 structures during S phase which in turn may stall replication forks. The processes of HR and translesion DNA synthesis (TLS) promote restart of stalled forks [50,51,52]. Indeed, in the absence of DOG-1, proteins implicated in the HR repair pathway (Rad51, BARD1) and TLS polymerases (polη, polk) are required to prevent G-tract deletions [36]. Embryonic lethality induced by loss of TLS polymerase REV-1 prevented researchers from studying its role in the maintenance of G/C tracts in DOG-1 mutants.

Recently, the role of polymerase Theta (POLQ-1) mediated alternative end-joining in promoting the repair of double-strand breaks caused by G4 induced arrest of replication forks in DOG-1 deficient *C. elegans* cells was addressed [53]. In the absence of dog-1 and polq-1, very large deletions occur at G4 sites. Polymerase Theta mediated alternative end-joining suppresses these large deletions and causes a narrow size deletion distribution between 50 and 300 base pairs at G4 sites in DOG-1 deficient cells. It remains to be seen if a similar mechanism operates in mammalian cells (see below). In other work, Youds et al. also showed that DOG-1 deficiency along with a loss of XPA-1 or CKU-80, orthologs of human XPA and Ku80, had no effect on G-tract deletions [36]. Thus, nucleotide excision repair or nonhomologous end-joining do not cause or prevent deletions at G/C tracts in the absence of DOG-1 [36].

## 5. Cross-Talk between FANCJ and Translesion Polymerases in Replication Past G4

Genetic studies using the chicken DT40 system have revealed valuable insights into cellular pathways of FANCJ-supported G4 replication important for epigenetic stability. FANCJ was shown to help coordinate leading and lagging strand synthesis to maintain chromatin structure necessary for normal recycling of histones to preserve epigenetic marks [38,39]. Whether this occurs during leading or lagging strand synthesis (or both) is debated. Evidence from the Niedzwiedz group demonstrated that FANCJ-deficient chicken cells accumulate small single-strand gaps that correspond to the size of gaps that arise in the absence of polymerase delta, which is required for primer extension during lagging strand synthesis [39]. Furthermore, the polarity of deletions with a G monotract at 3′ junctions in dog-1 mutants suggests that the helicase unwinds G4 on the lagging strand template during DNA replication [34]. Flipping the coin, the Sale group presented evidence to implicate FANCJ in resolving G4 in the leading strand template [38]. Two pathways were proposed to maintain epigenetic stability at G4, one that was dependent on the TLS polymerase REV1, which is implicated in replication of a G4 motif residing in the leading strand template [54]. Remarkably, a single G4 motif in the leading strand template located as far away as 4 kilobases from the transcription start site of an actively expressed locus is affected by REV1 deficiency [55].

**Figure 2:**
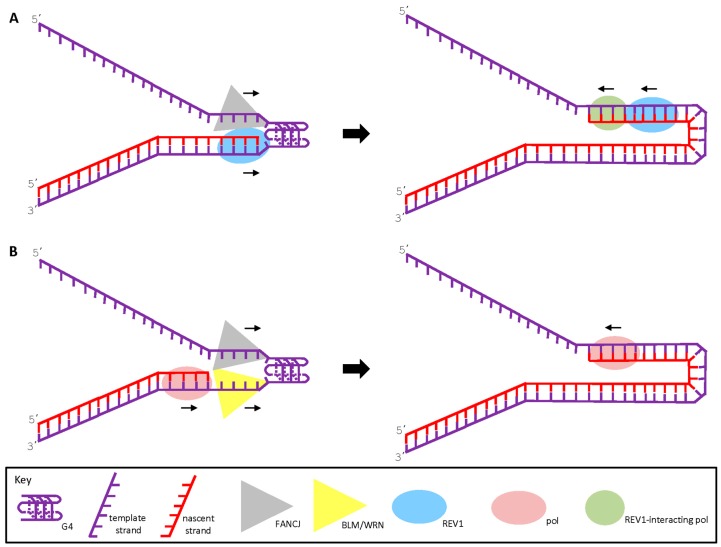
Proposed models for how FANCJ protein interactions provide a mechanistic opportunity to resolve G4 structures to enable smooth DNA synthesis. (**A**) FANCJ interacts with the TLS polymerase REV1 at the site of a G4 structure, enabling it to catalyze DNA synthesis past the G4 obstacle. A REV-1 interacting polymerase is likely to also be involved; (**B**) FANCJ interacts with the BLM (or WRN) helicase to efficiently resolve the G4 DNA structure, making the single-strand template accessible to a replicative DNA polymerase. For either model, the role of FANCJ may prevail for leading or lagging strand DNA synthesis. See text for details.

REV1 is known to serve as a scaffolding protein that helps to recruit other TLS polymerases to assist in replicating DNA past sites of DNA damage in a Proliferating cell nuclear antigen (PCNA)-dependent manner [56,57]. Thus, it will be informative to identify from the Xenopus cell-free system the relevant REV1 interacting TLS polymerases involved in G4 bypass. The PCNA interaction with REV1 is mediated by REV1 BRCT repeat domains that are required for REV1 recruitment to replication foci [58]. A distinct possibility is that human FANCJ, a protein that interacts with the BRCT repeats of BRCA1 [17], physically binds to REV1 in a tradeoff with PCNA and facilitates DNA synthesis past the G4 (Figure 2). In this scenario, a single-stranded tract in the lagging strand template, or one created in the leading strand template when DNA unwinding by the replicative Minichromosome maintenance protein (MCM) complex becomes transiently uncoupled from DNA synthesis at the fork, provides the loading dock for FANCJ to unwind the G4 structure with its characteristic 5′ to 3′ directionality. Going back to the putative interaction of FANCJ with REV1, a recent biochemical study suggests that REV1 itself has the capacity to efficiently bind a G-quadruplex, and can disrupt G4 DNA or prevent it from refolding [59]. However, REV1 acting alone displays a reduced catalytic efficiency to insert dCMP opposite tetrad-guanines, suggesting that other TLS polymerases or G4-interacting proteins such as helicases may come into play to enhance the process. We favor the hypothesis that FANCJ may be a prominent player that irons out G4 for template-directed DNA synthesis, acting in concert with REV1 and REV1-interacting TLS polymerases, and perhaps replicative polymerases in other settings (see below). Depletion of the TLS polymerases eta and kappa renders human cancer cells sensitive to TMS, suggesting that these enzymes are good candidates for overcoming non-B form DNA structures like G4 [60]. Interestingly, disruption of the BRCA1-FANCJ interaction releases FANCJ to promote bypass of ICL-induced DNA damage by polymerase eta in a MLH1-dependent manner as opposed to mediating DNA repair by HR [61]. It remains to be seen if a similar mechanism operates for FANCJ to promote DNA synthesis past G4 barriers. To our knowledge, there has not been any report of a direct physical interaction of FANCJ with a translesion DNA polymerase.

Based on work from *C. elegans*, another prominent pathway that suppresses genomic instability at G4 DNA sites is mediated by the translesion DNA polymerase theta. Elegant studies from the Tijsterman lab showed that replication-associated breaks occurring at predicted G4-forming sequences are repaired by polymerase theta-supported end-joining [53,62]. Although this mechanism is error-prone, it protects the nematode’s genome from gross chromosomal rearrangements. A model is proposed that helps to explain the non-symmetric deletions of *C. elegans* dog-1 mutants in which theta-mediated end-joining of G4-induced DNA breaks is responsible [63]. It is of great interest if the human homolog of theta, known as DNA polymerase Q [64], plays an analogous role in repair of G4-induced DNA damage. Evidence from worm studies suggest that a single G4 causes a persistent replication block that elicits the alternative end-joining pathway resulting in multiple genomic deletions at one genomic location; however, the frequency of characteristic small-sized deletions near the G4 motif were not exacerbated in dog-1 mutants exposed to a G4-stabilizing ligand [65]. Nonetheless, it is reasonable to propose that human FANCJ and polymerase Q collaborate to repair G4-associated DNA breaks, but this remains to be seen. Just as there has been much interest in the mechanisms whereby specialized DNA polymerases act to replicate structured DNA [66], their coordination with other DNA metabolic enzymes such as helicases is likely to be important.

## 6. Cross-Talk between FANCJ and RecQ Helicases in Replication Past G4

The other FANCJ-dependent pathway to maintain epigenetic stability involves the RecQ helicases WRN or BLM defective in Werner syndrome and Bloom syndrome, respectively [38]. It is predicted that FANCJ would operate on the opposite side of the G4 structure approached by either REV1 or the WRN or BLM helicases, which require a 3′ single-stranded region to unwind an adjacent G4 [45,46,47]. However, FANCJ protein interactions may influence the spatial architecture of how FANCJ helps to overcome replication pausing at G4 (Figure 2). FANCJ was already reported to interact physically and functionally with BLM helicase [67], suggesting the two interacting DNA repair helicases may facilitate smooth DNA synthesis past the leading or lagging strand G4 structure during replication. Conceivably, BLM would translocate on the opposite strand as FANCJ, and together the two helicases collaborate to efficiently resolve the G4 allowing the unfolded G-rich sequence to be copied.

A potential role of WRN in G4 metabolism was suggested by an in vitro study of Kamath-Loeb et al. which showed that an interaction between WRN and polymerase delta facilitates DNA synthesis past a G4 obstacle in a DNA template [68]. WRN (or BLM) may also play a role in the aforementioned TLS pathway to replicate past a G4 obstacle (Figure 2). Previously, WRN was shown to functionally interact with TLS DNA polymerases on lesion-free and lesion-containing DNA templates in primer extension assays; however, G4-nested templates were not evaluated [69].

## 7. A Role of FANCJ to Suppress Microsatellite Instability

Microsatellite repeat sequences, typically 1–10 base pairs, are prone to form secondary DNA structures (hairpins, triplexes) during replication, resulting in template slippage that can lead to expansions or contractions [2]. Fancj^−/−^ mouse embryonic fibroblasts (MEFS) were found to display microsatellite instability that was not observed in Fancd2^−/−^ MEFs [41]. Sequence analysis of the microsatellites from Fancj^−/−^ MEFs showed a constellation of contractions, expansions, and more complex DNA sequences, suggesting that template slippage during replication occurred frequently in the Fancj^−/−^ MEFs. Microsatellite instability was also observed in FA-J patient-derived cells or CRISPR-derived Fancj^−/−^ U2 OS cells, and the instability was even greater in those cells exposed to the DNA polymerase inhibitor aphidicolin [41]. Presumably, replication stress induced by aphidicolin would lead to unusual replication fork configurations characterized by a more single-strand character, which would be prone to forming secondary structure. Replication tract analysis of Fancj^−/−^ and Fancj^+/+^ MEFs confirmed that murine FANCJ is important for normal replication fork progression, particularly when forks are stalled by aphidicolin [41]. Similarly, human FANCJ-deficient cells were reported to be sensitive to hydroxyurea, an agent that also inhibits replication, only by depleting the nucleotide pool [67]. The elevated incidence and early onset of lymphomas in Fancj^−/−^ mice, which is not typically observed in other FA mutant mouse models, led the authors to suggest a unique role of FANCJ in cancer suppression to preserve genomic stability by resolving secondary DNA structures, thereby inhibiting microsatellite instability [41]. The finding that FANCJ deficiency causes general microsatellite instability under conditions of replication stress was also reported by the Leffak lab [70]. Loss-of-function germline mutations in other FA proteins did not cause the microsatellite instability observed in FANCJ-deficient cells, indicating that FANCJ operates outside the classic FA pathway to maintain microsatellite structure.

## 8. Involvement of FANCJ in Spermatogenesis

Studies using another Fancj^−/−^ mouse model suggest an important role of the helicase in gametogenesis to enable spermatogonial proliferation in the developing testis and appropriate processing of crossovers between homologous chromosomes during prophase I of meiosis [71]. The elevated crossovers in Fancj-deficient mice are likely to arise from defective double-strand break repair. Further studies are necessary to characterize the specific DNA recombination intermediates acted upon by FANCJ and how these events are orchestrated with other proteins in meiosis. FANCJ may help to suppress aberrant processing of three-stranded or four-stranded mobile DNA joint molecules that originate via HR at double-strand breaks, which, if left unresolved, destabilize the genome and contribute to spermatogonial cell depletion.

## 9. Potential Influence of FANCJ and G4-Interacting Helicases in Transcriptional Regulation and Telomere Capping

The ability of FANCJ to resolve G4 DNA is likely to extend beyond DNA replication and affect other cellular processes. G4 motifs are prominent in promoters and first introns, suggesting that G4 may influence transcriptional regulation [6,72]. The G4 motifs may upregulate or downregulate gene expression depending on their location. Gene expression may be blocked with the G4 motifs residing near or within the promoter region, whereas expression may possibly be enhanced with the G4 motifs located in the non-coding DNA strand to help maintain the open conformation of the coding DNA strand; however gene pausing and activation are likely complex with multiple mechanisms coming into play [73]. Interestingly, DNA sequences predicted to form G4 are enriched in oncogenes, whereas they are found relatively lower in abundance in tumor suppressor genes [6], suggesting their differential involvement in gene regulation in cancer cells versus normal cells; however, further study in this area is required.

For transcriptional regulation, DNA helicases have moved to the forefront with the discovery that the XPB and XPD helicases of the general transcription factor (TF) IIH are enriched in their association with predicted genomic G4 DNA, including the transcriptional start sites of highly transcribed genes [74]. In another study, BLM regulation of gene expression correlated with the presence of G4 motifs at transcriptional start sites and first introns [75]. This work builds upon earlier work showing altered expression of genes associated with G4-forming sequences in cells derived from Bloom syndrome and Werner syndrome [76]. In yet another study, RECQ1 was found to bind G4 motifs in the promoters of genes that were downregulated by RECQ1 silencing [77]. Most recently, WRN was reported to bind a set of G4 motifs distinct from that of BLM in human cells [78]. Despite these pieces of evidence, it is unclear if G4 DNA unwinding versus binding by all these DNA helicases is important for their roles in transcriptional regulation in human cells, given that only human BLM and WRN have been formally tested and shown to unwind G4 DNA [45,46,47]. It will be important to assess the role of FANCJ in regulating transcription initiation by its action at G4-laden promoters and associated elements. Given FANCJ’s status as a tumor suppressor [79], this issue is particularly relevant for expression of oncogenes where G4 motifs are highly abundant [6,80,81]. However, to our knowledge it is not yet known if FANCJ plays different gene regulatory roles in normal versus cancer cells.

To accurately assess the biological importance of G4-forming sequences predicted by algorithms, it will be necessary to acquire in vitro and in vivo evidence that such G-rich sequences do indeed form G4 structures and characterize their molecular architecture. For example, several biophysical studies demonstrate that the loop sequence and length as well as other factors (cation choice and concentration or molecular crowding agents) exert marked effects on G4 stability and topology [82,83,84]. An even greater challenge is to determine the architecture of a G4 structure in living cells. Several useful tools including the first generation of G4 antibodies have been developed [40,85], and may be useful for this purpose. Once the topology of the G4 is established, it will be informative to ascertain the relationship between G4 topology and prevalence in the human genome as well as relevance for biological processes such as transcriptional regulation or telomere maintenance. Moreover, it will be important to characterize the substrate preference of FANCJ and other G4-resolving helicases. Our recent work demonstrated that FANCJ is unique among Fe-S helicases tested in its ability to efficiently unwind intramolecular G4 DNA substrates in vitro [28]. The human G4 Resolvase 1 (G4R1) helicase preferentially binds to a parallel unimolecular G4 substrate [84], whereas human DDX11 strongly prefers to unwind anti-parallel intermolecular G4 substrates [86]. Thus, differences in the G4 substrate specificity among helicases are likely to exist that will be important for their biological roles.

A potential role of FANCJ in resolving G4 DNA structures that arise from the G-rich telomeric repeats at chromosome ends should not be overlooked. Although the DNA helicases PIF1 and RTEL-1 are thought to play prominent roles in telomere G4 DNA metabolism (reviewed in [87,88]), FANCJ was localized to telomeres in cells that operate by the alternate lengthening of telomeres (ALT) pathway [89]. Biochemical evidence suggests that FANCJ can remodel shelterin proteins bound to telomeric DNA in a Replication Protein A (RPA)-dependent manner [90]; however, a role of FANCJ in telomeric G4 DNA resolution remains to be elucidated.

## 10. Conclusions

The importance of processive DNA replication has been reinforced by studies over a number of years; however, this concept has largely developed from experiments in which cells are exposed to agents that induce DNA damage or interfere with the ability of DNA polymerase to incorporate nucleotides during nascent DNA synthesis (reviewed in [91,92,93]). A heightened awareness of the biological effects of naturally occurring structured DNA on replication-associated events is beginning to emerge. Given the vast abundance of predicted G4 forming sequences in the human genome, G4 DNA is likely to be highly influential for a variety of cellular processes including telomere maintenance, gene regulation, and genetic recombination in addition to DNA replication.

New evidence has implicated G4 DNA structures to play a role in cancer. Predicted G4-forming sequences associated with abnormal hypomethylation are enriched in the proximity of breakpoints associated with somatic alterations in cancer, leading the authors to propose that the mutagenic potential of G4 DNA structures is affected by epigenetic status [94]. A more recent study demonstrated that predicted G4-forming DNA sequences are enriched at human copy number variation breakpoints that play a role in chromosome fragile sites prevalent in patients with neurodevelopmental disorders [95]. Further work in this area will potentially lead to an understanding of how DNA secondary structure affects susceptibility to double strand breaks and errors in DNA replication.

The influence of DNA secondary structure on checkpoint activation and the role of helicases in this capacity have not been investigated to a great extent. The Leffak lab examined replication fork stalling and checkpoint activation triggered by a polypurine-polypyrimidine tract from the human polycystic kidney disease that has the potential to form triplex and G-quadruplex DNA structures [96]. Based on their results from chromatin immunoprecipitation experiments with HeLa cell extracts, the authors concluded that non-B DNA structure in the purine-rich lagging strand template drove checkpoint activation via the ATR-Chk1 signaling pathway. In subsequent work, the Leffak [70] and Boulton [41] labs determined that FANCJ plays an important role to suppress microsatellite instability, which becomes even more apparent under conditions of replication stress. FANCJ has been found to mediate ATR checkpoint signaling via its interaction with TopBP1 following replication stalling induced by cellular exposure to hydroxyurea, an agent that causes depletion of the nucleotide pool [97]. Cells deficient in FANCJ were significantly attenuated in loading of RPA onto chromatin, a critical event in the signal transduction pathway. Further work is required to assess if FANCJ’s resolution of secondary DNA structure at stalled forks is important for enhanced RPA loading to facilitate the replication checkpoint.

Although many questions remain, the development of eukaryotic models like that offered by the Xenopus cell-free system as well as avian, nematode, and human cell-based genetics should continue to advance our understanding of G4 biology. Experimental evidence indicates that FANCJ and other helicases play a highly significant role in the resolution of G4 and other DNA structures important in cellular nucleic acid metabolism. In light of recent progress and our improved understanding of genome stability, G4 wrinkles, which are likely to be highly abundant, may provide a molecular target for anti-cancer drugs that impede DNA replication in rapidly dividing cancer cells [98]. Therefore, it will be of great interest to ascertain if FANCJ and other helicases (e.g., WRN [99,100,101]) are suitable for modulation in emerging cancer therapies.

## References

[1] Leon-Ortiz A.M., Svendsen J., Boulton S.J. (2014). Metabolism of DNA secondary structures at the eukaryotic replication fork. DNA Repair.

[2] Wang G., Vasquez K.M. (2014). Impact of alternative DNA structures on DNA damage, DNA repair, and genetic instability. DNA Repair.

[3] Gellert M., Lipsett M.N., Davies D.R. (1962). Helix formation by guanylic acid. Proc. Natl. Acad. Sci. USA.

[4] Sen D., Gilbert W. (1988). Formation of parallel four-stranded complexes by guanine-rich motifs in DNA and its implications for meiosis. Nature.

[5] Bedrat A., Lacroix L., Mergny J.L. (2016). Re-evaluation of G-quadruplex propensity with G4Hunter. Nucleic Acids Res..

[6] Eddy J., Maizels N. (2006). Gene function correlates with potential for G4 DNA formation in the human genome. Nucleic Acids Res..

[7] Huppert J.L., Balasubramanian S. (2005). Prevalence of quadruplexes in the human genome. Nucleic Acids Res..

[8] Todd A.K., Johnston M., Neidle S. (2005). Highly prevalent putative quadruplex sequence motifs in human DNA. Nucleic Acids Res..

[9] Bharti S.K., Sommers J.A., Zhou J., Kaplan D.L., Spelbrink J.N., Mergny J.L., Brosh R.M. (2014). DNA sequences proximal to human mitochondrial DNA deletion breakpoints prevalent in human disease form G-quadruplexes, a class of DNA structures inefficiently unwound by the mitochondrial replicative Twinkle helicase. J. Biol. Chem..

[10] Dong D.W., Pereira F., Barrett S.P., Kolesar J.E., Cao K., Damas J., Yatsunyk L.A., Johnson F.B., Kaufman B.A. (2014). Association of G-quadruplex forming sequences with human mtDNA deletion breakpoints. BMC Genom..

[11] Murat P., Balasubramanian S. (2014). Existence and consequences of G-quadruplex structures in DNA. Curr. Opin. Genet. Dev..

[12] Rhodes D., Lipps H.J. (2015). G-quadruplexes and their regulatory roles in biology. Nucleic Acids Res..

[13] Maizels N. (2015). G4-associated human diseases. EMBO Rep..

[14] Wu Y., Brosh R.M. (2010). G-quadruplex nucleic acids and human disease. FEBS J..

[15] Brosh R.M. (2013). DNA helicases involved in DNA repair and their roles in cancer. Nat. Rev. Cancer.

[16] Mendoza O., Bourdoncle A., Boule J.B., Brosh R.M., Mergny J.L. (2016). G-quadruplexes and helicases. Nucleic Acids Res..

[17] Cantor S.B., Bell D.W., Ganesan S., Kass E.M., Drapkin R., Grossman S., Wahrer D.C., Sgroi D.C., Lane W.S., Haber D.A. (2001). BACH1, a novel helicase-like protein, interacts directly with BRCA1 and contributes to its DNA repair function. Cell.

[18] Rafnar T., Gudbjartsson D.F., Sulem P., Jonasdottir A., Sigurdsson A., Jonasdottir A., Besenbacher S., Lundin P., Stacey S.N., Gudmundsson J. (2011). Mutations in BRIP1 confer high risk of ovarian cancer. Nat. Genet..

[19] Seal S., Thompson D., Renwick A., Elliott A., Kelly P., Barfoot R., Chagtai T., Jayatilake H., Ahmed M., Spanova K. (2006). Truncating mutations in the Fanconi anemia J gene BRIP1 are low-penetrance breast cancer susceptibility alleles. Nat. Genet..

[20] Levitus M., Waisfisz Q., Godthelp B.C., de Vries Y., Hussain S., Wiegant W.W., Elghalbzouri-Maghrani E., Steltenpool J., Rooimans M.A., Pals G. (2005). The DNA helicase BRIP1 is defective in Fanconi anemia complementation group J. Nat. Genet..

[21] Levran O., Attwooll C., Henry R.T., Milton K.L., Neveling K., Rio P., Batish S.D., Kalb R., Velleuer E., Barral S. (2005). The BRCA1-interacting helicase BRIP1 is deficient in Fanconi anemia. Nat. Genet..

[22] Litman R., Peng M., Jin Z., Zhang F., Zhang J., Powell S., Andreassen P.R., Cantor S.B. (2005). BACH1 is critical for homologous recombination and appears to be the Fanconi anemia gene product FANCJ. Cancer Cell.

[23] Rudolf J., Makrantoni V., Ingledew W.J., Stark M.J., White M.F. (2006). The DNA Repair Helicases XPD and FancJ have essential iron-sulfur domains. Mol. Cell.

[24] Wu Y., Sommers J.A., Suhasini A.N., Leonard T., Deakyne J.S., Mazin A.V., Shin-Ya K., Kitao H., Brosh R.M. (2010). Fanconi anemia Group J mutation abolishes its DNA repair function by uncoupling DNA translocation from helicase activity or disruption of protein-DNA complexes. Blood.

[25] Brosh R.M., Cantor S.B. (2014). Molecular and cellular functions of the FANCJ DNA helicase defective in cancer and in Fanconi anemia. Front. Genet..

[26] Cantor S.B., Nayak S. (2016). FANCJ at the FORK. Mutat. Res..

[27] Wu Y., Shin-Ya K., Brosh R.M. (2008). FANCJ helicase defective in Fanconia Anemia and breast cancer unwinds G-quadruplex DNA to defend genomic stability. Mol. Cell Biol..

[28] Bharti S.K., Sommers J.A., George F., Kuper J., Hamon F., Shin-Ya K., Teulade-Fichou M.P., Kisker C., Brosh R.M. (2013). Specialization among iron-sulfur cluster helicases to resolve G-quadruplex DNA structures that threaten genomic stability. J. Biol. Chem..

[29] London T.B., Barber L.J., Mosedale G., Kelly G.P., Balasubramanian S., Hickson I.D., Boulton S.J., Hiom K. (2008). FANCJ is a structure-specific DNA helicase associated with the maintenance of genomic G/C tracts. J. Biol. Chem..

[30] Kuper J., Wolski S.C., Michels G., Kisker C. (2012). Functional and structural studies of the nucleotide excision repair helicase XPD suggest a polarity for DNA translocation. EMBO J..

[31] Pugh R.A., Wu C.G., Spies M. (2012). Regulation of translocation polarity by helicase domain 1 in SF2B helicases. EMBO J..

[32] Castillo Bosch P., Segura-Bayona S., Koole W., van Heteren J.T., Dewar J.M., Tijsterman M., Knipscheer P. (2014). FANCJ promotes DNA synthesis through G-quadruplex structures. EMBO J..

[33] Raghunandan M., Chaudhury I., Kelich S.L., Hanenberg H., Sobeck A. (2015). FANCD2, FANCJ and BRCA2 cooperate to promote replication fork recovery independently of the Fanconi Anemia core complex. Cell Cycle.

[34] Cheung I., Schertzer M., Rose A., Lansdorp P.M. (2002). Disruption of dog-1 in Caenorhabditis elegans triggers deletions upstream of guanine-rich DNA. Nat. Genet..

[35] Kruisselbrink E., Guryev V., Brouwer K., Pontier D.B., Cuppen E., Tijsterman M. (2008). Mutagenic capacity of endogenous G4 DNA underlies genome instability in FANCJ-defective *C. elegans*. Curr. Biol..

[36] Youds J.L., O’Neil N.J., Rose A.M. (2006). Homologous recombination is required for genome stability in the absence of DOG-1 in Caenorhabditis elegans. Genetics.

[37] Kitao H., Nanda I., Sugino R.P., Kinomura A., Yamazoe M., Arakawa H., Schmid M., Innan H., Hiom K., Takata M. (2011). FancJ/Brip1 helicase protects against genomic losses and gains in vertebrate cells. Genes Cells.

[38] Sarkies P., Murat P., Phillips L.G., Patel K.J., Balasubramanian S., Sale J.E. (2012). FANCJ coordinates two pathways that maintain epigenetic stability at G-quadruplex DNA. Nucleic Acids Res..

[39] Schwab R.A., Nieminuszczy J., Shin-Ya K., Niedzwiedz W. (2013). FANCJ couples replication past natural fork barriers with maintenance of chromatin structure. J. Cell Biol..

[40] Henderson A., Wu Y., Huang Y.C., Chavez E.A., Platt J., Johnson F.B., Brosh R.M., Sen D., Lansdorp P.M. (2013). Detection of G-quadruplex DNA in mammalian cells. Nucleic Acids Res..

[41] Matsuzaki K., Borel V., Adelman C.A., Schindler D., Boulton S.J. (2015). FANCJ suppresses microsatellite instability and lymphomagenesis independent of the Fanconi anemia pathway. Genes Dev..

[42] Vannier J.B., Sandhu S., Petalcorin M.I., Wu X., Nabi Z., Ding H., Boulton S.J. (2013). RTEL1 is a replisome-associated helicase that promotes telomere and genome-wide replication. Science.

[43] Ding H., Schertzer M., Wu X., Gertsenstein M., Selig S., Kammori M., Pourvali R., Poon S., Vulto I., Chavez E. (2004). Regulation of murine telomere length by Rtel: An essential gene encoding a helicase-like protein. Cell.

[44] Vannier J.B., Pavicic-Kaltenbrunner V., Petalcorin M.I., Ding H., Boulton S.J. (2012). RTEL1 dismantles T loops and counteracts telomeric G4-DNA to maintain telomere integrity. Cell.

[45] Fry M., Loeb L.A. (1999). Human werner syndrome DNA helicase unwinds tetrahelical structures of the fragile X syndrome repeat sequence d(CGG)n. J. Biol. Chem..

[46] Mohaghegh P., Karow J.K., Brosh R.M., Bohr V.A., Hickson I.D. (2001). The Bloom’s and Werner’s syndrome proteins are DNA structure-specific helicases. Nucleic Acids Res..

[47] Sun H., Karow J.K., Hickson I.D., Maizels N. (1998). The Bloom’s syndrome helicase unwinds G4 DNA. J. Biol. Chem..

[48] Barber L.J., Youds J.L., Ward J.D., McIlwraith M.J., O’Neil N.J., Petalcorin M.I., Martin J.S., Collis S.J., Cantor S.B., Auclair M. (2008). RTEL1 maintains genomic stability by suppressing homologous recombination. Cell.

[49] Tarailo-Graovac M., Wong T., Qin Z., Flibotte S., Taylor J., Moerman D.G., Rose A.M., Chen N. (2015). Spectrum of variations in dog-1/FANCJ and mdf-1/MAD1 defective Caenorhabditis elegans strains after long-term propagation. BMC Genom..

[50] Lehmann A.R. (2002). Replication of damaged DNA in mammalian cells: New solutions to an old problem. Mutat. Res..

[51] Michel B., Flores M.J., Viguera E., Grompone G., Seigneur M., Bidnenko V. (2001). Rescue of arrested replication forks by homologous recombination. Proc. Natl. Acad. Sci. USA.

[52] Saleh-Gohari N., Bryant H.E., Schultz N., Parker K.M., Cassel T.N., Helleday T. (2005). Spontaneous homologous recombination is induced by collapsed replication forks that are caused by endogenous DNA single-strand breaks. Mol. Cell Biol..

[53] Koole W., van Schendel R., Karambelas A.E., van Heteren J.T., Okihara K.L., Tijsterman M. (2014). A Polymerase Theta-dependent repair pathway suppresses extensive genomic instability at endogenous G4 DNA sites. Nat. Commun..

[54] Sarkies P., Reams C., Simpson L.J., Sale J.E. (2010). Epigenetic instability due to defective replication of structured DNA. Mol. Cell.

[55] Schiavone D., Guilbaud G., Murat P., Papadopoulou C., Sarkies P., Prioleau M.N., Balasubramanian S., Sale J.E. (2014). Determinants of G quadruplex-induced epigenetic instability in REV1-deficient cells. EMBO J..

[56] Guo C., Fischhaber P.L., Luk-Paszyc M.J., Masuda Y., Zhou J., Kamiya K., Kisker C., Friedberg E.C. (2003). Mouse Rev1 protein interacts with multiple DNA polymerases involved in translesion DNA synthesis. EMBO J..

[57] Ross A.L., Simpson L.J., Sale J.E. (2005). Vertebrate DNA damage tolerance requires the C-terminus but not BRCT or transferase domains of REV1. Nucleic Acids Res..

[58] Guo C., Sonoda E., Tang T.S., Parker J.L., Bielen A.B., Takeda S., Ulrich H.D., Friedberg E.C. (2006). REV1 protein interacts with PCNA: Significance of the REV1 BRCT domain in vitro and in vivo. Mol. Cell.

[59] Eddy S., Ketkar A., Zafar M.K., Maddukuri L., Choi J.Y., Eoff R.L. (2014). Human Rev1 polymerase disrupts G-quadruplex DNA. Nucleic Acids Res..

[60] Betous R., Rey L., Wang G., Pillaire M.J., Puget N., Selves J., Biard D.S., Shin-Ya K., Vasquez K.M., Cazaux C. (2009). Role of TLS DNA polymerases eta and kappa in processing naturally occurring structured DNA in human cells. Mol. Carcinog..

[61] Xie J., Litman R., Wang S., Peng M., Guillemette S., Rooney T., Cantor S.B. (2010). Targeting the FANCJ-BRCA1 interaction promotes a switch from recombination to poleta-dependent bypass. Oncogene.

[62] Roerink S.F., van Schendel R., Tijsterman M. (2014). A Polymerase Theta-mediated end joining of replication-associated DNA breaks in *C. elegans*. Genome Res..

[63] Van Kregten M., Tijsterman M. (2014). The repair of G-quadruplex-induced DNA damage. Exp. Cell Res..

[64] Seki M., Marini F., Wood R.D. (2003). POLQ (Pol theta), a DNA polymerase and DNA-dependent ATPase in human cells. Nucleic Acids Res..

[65] Lemmens B., van S.R., Tijsterman M. (2015). Mutagenic consequences of a single G-quadruplex demonstrate mitotic inheritance of DNA replication fork barriers. Nat. Commun..

[66] Wickramasinghe C.M., Arzouk H., Frey A., Maiter A., Sale J.E. (2015). Contributions of the specialised DNA polymerases to replication of structured DNA. DNA Repair.

[67] Suhasini A.N., Rawtani N.A., Wu Y., Sommers J.A., Sharma S., Mosedale G., North P.S., Cantor S.B., Hickson I.D., Brosh R.M. (2011). Interaction between the helicases genetically linked to Fanconi anemia group J and Bloom’s syndrome. EMBO J..

[68] Kamath-Loeb A.S., Loeb L.A., Johansson E., Burgers P.M., Fry M. (2001). Interactions between the Werner syndrome helicase and DNA Polymerase {delta} specifically facilitate copying of tetraplex and hairpin structures of the d(CGG) trinucleotide repeat sequence. J. Biol. Chem..

[69] Kamath-Loeb A.S., Lan L., Nakajima S., Yasui A., Loeb L.A. (2007). Werner syndrome protein interacts functionally with translesion DNA polymerases. Proc. Natl. Acad. Sci. USA.

[70] Barthelemy J., Hanenberg H., Leffak M. (2016). FANCJ is essential to maintain microsatellite structure genome-wide during replication stress. Nucleic Acids Res..

[71] Sun X., Brieno-Enriquez M.A., Cornelius A., Modzelewski A.J., Maley T.T., Campbell-Peterson K.M., Holloway J.K., Cohen P.E. (2016). FancJ (Brip1) loss-of-function allele results in spermatogonial cell depletion during embryogenesis and altered processing of crossover sites during meiotic prophase I in mice. Chromosoma.

[72] Huppert J.L., Balasubramanian S. (2007). G-quadruplexes in promoters throughout the human genome. Nucleic Acids Res..

[73] Eddy J., Vallur A.C., Varma S., Liu H., Reinhold W.C., Pommier Y., Maizels N. (2011). G4 motifs correlate with promoter-proximal transcriptional pausing in human genes. Nucleic Acids Res..

[74] Gray L.T., Vallur A.C., Eddy J., Maizels N. (2014). G quadruplexes are genomewide targets of transcriptional helicases XPB and XPD. Nat. Chem. Biol..

[75] Nguyen G.H., Tang W., Robles A.I., Beyer R.P., Gray L.T., Welsh J.A., Schetter A.J., Kumamoto K., Wang X.W., Hickson I.D. (2014). Regulation of gene expression by the BLM helicase correlates with the presence of G-quadruplex DNA motifs. Proc. Natl. Acad. Sci. USA.

[76] Johnson J.E., Cao K., Ryvkin P., Wang L.S., Johnson F.B. (2010). Altered gene expression in the Werner and Bloom syndromes is associated with sequences having G-quadruplex forming potential. Nucleic Acids Res..

[77] Li X.L., Lu X., Parvathaneni S., Bilke S., Zhang H., Thangavel S., Vindigni A., Hara T., Zhu Y., Meltzer P.S. (2014). Identification of RECQ1-regulated transcriptome uncovers a role of RECQ1 in regulation of cancer cell migration and invasion. Cell Cycle.

[78] Tang W., Robles A.I., Beyer R.P., Gray L.T., Nguyen G.H., Oshima J., Maizels N., Harris C.C., Monnat R.J. (2016). The Werner syndrome RECQ helicase targets G4 DNA in human cells to modulate transcription. Hum. Mol. Genet..

[79] Cantor S.B., Guillemette S. (2011). Hereditary breast cancer and the BRCA1-associated FANCJ/BACH1/BRIP1. Future Oncol..

[80] Brown R.V., Danford F.L., Gokhale V., Hurley L.H., Brooks T.A. (2011). Demonstration that drug-targeted down-regulation of MYC in non-Hodgkins lymphoma is directly mediated through the promoter G-quadruplex. J. Biol. Chem..

[81] Duquette M.L., Huber M.D., Maizels N. (2007). G-rich proto-oncogenes are targeted for genomic instability in B-cell lymphomas. Cancer Res..

[82] Guedin A., Gros J., Alberti P., Mergny J.L. (2010). How long is too long? Effects of loop size on G-quadruplex stability. Nucleic Acids Res..

[83] Hazel P., Huppert J., Balasubramanian S., Neidle S. (2004). Loop-length-dependent folding of G-quadruplexes. J. Am. Chem. Soc..

[84] Tippana R., Xiao W., Myong S. (2014). G-quadruplex conformation and dynamics are determined by loop length and sequence. Nucleic Acids Res..

[85] Biffi G., Tannahill D., McCafferty J., Balasubramanian B. (2013). Quantitative visulaization of DNA G-quadruplex structures in human cells. Nat. Chem..

[86] Wu Y., Sommers J.A., Khan I., De Winter J.P., Brosh R.M. (2012). Biochemical characterization of Warsaw breakage syndrome helicase. J. Biol. Chem..

[87] Paeschke K., McDonald K.R., Zakian V.A. (2010). Telomeres: Structures in need of unwinding. FEBS Lett..

[88] Vannier J.B., Sarek G., Boulton S.J. (2014). RTEL1: Functions of a disease-associated helicase. Trends Cell Biol..

[89] Dejardin J., Kingston R.E. (2009). Purification of proteins associated with specific genomic Loci. Cell.

[90] Sommers J.A., Banerjee T., Hinds T., Wold M.S., Lei M., Brosh R.M. (2014). Novel function of the Fanconi Anemia Group J or RECQ1 helicase to disrupt protein-DNA complexes in a Replication Protein A-stimulated Manner. J. Biol. Chem..

[91] Branzei D., Foiani M. (2010). Maintaining genome stability at the replication fork. Nat. Rev. Mol. Cell Biol..

[92] Budzowska M., Kanaar R. (2009). Mechanisms of dealing with DNA damage-induced replication problems. Cell Biochem. Biophys..

[93] Lambert S., Carr A.M. (2013). Impediments to replication fork movement: Stabilisation, reactivation and genome instability. Chromosoma.

[94] De S., Michor F. (2011). DNA secondary structures and epigenetic determinants of cancer genome evolution. Nat. Struct. Mol. Biol..

[95] Bose P., Hermetz K.E., Conneely K.N., Rudd M.K. (2014). Tandem repeats and G-rich sequences are enriched at human CNV breakpoints. PLoS ONE.

[96] Liu G., Myers S., Chen X., Bissler J.J., Sinden R.R., Leffak M. (2012). Replication fork stalling and checkpoint activation by a PKD1 locus mirror repeat polypurine-polypyrimidine (Pu-Py) tract. J. Biol. Chem..

[97] Gong Z., Kim J.E., Leung C.C., Glover J.N., Chen J. (2010). BACH1/FANCJ acts with TopBP1 and participates early in DNA replication checkpoint control. Mol. Cell.

[98] Hale T.K., Norris G.E., Jameson G.B., Filichev V.V. (2014). Helicases, G4-DNAs, and drug design. ChemMedChem.

[99] Aggarwal M., Banerjee T., Sommers J.A., Iannascoli C., Pichierri P., Shoemaker R.H., Brosh R.M. (2013). Werner syndrome helicase has a critical role in DNA damage responses in the absence of a functional Fanconi Anemia pathway. Cancer Res..

[100] Aggarwal M., Sommers J.A., Shoemaker R.H., Brosh R.M. (2011). Inhibition of helicase activity by a small molecule impairs Werner syndrome helicase (WRN) function in the cellular response to DNA damage or replication stress. Proc. Natl. Acad. Sci. USA.

[101] Aggarwal M., Banerjee T., Sommers J.A., Brosh R.M. (2013). Targeting an Achilles’ heel of cancer with a WRN helicase inhibitor. Cell Cycle.

